# Direct Measurement of Protein Corona Thickness on Nanomaterials Using Dual‐Color Stochastic Optical Reconstruction Microscopy (STORM)

**DOI:** 10.1002/smtd.202502394

**Published:** 2026-05-11

**Authors:** Bradley T. Cech, Sophie M. Sullivan, Christopher C. Landry

**Affiliations:** ^1^ Department of Chemistry University of Vermont Burlington Vermont USA

**Keywords:** bio‐nano interface, nanoparticles, protein corona, STORM imaging, super‐resolution microscopy

## Abstract

Nanoparticles introduced to biological media rapidly adsorb proteins to form a protein corona that alters their surface identity and governs biological interactions. While protein corona composition has been extensively studied, its spatial structure, particularly thickness at the single‐particle level, remains poorly resolved. Here, we establish and validate a strategy using dual‐color stochastic optical reconstruction microscopy (STORM) for the direct measurement of hard corona thickness on individual nanoparticles. The nanoparticle surface and retained protein corona were labeled with spectrally distinct fluorophores, allowing the radial separation between the nanoparticle and adsorbed protein layer to be determined from single‐particle localization distributions. Applied to dense silica, mesoporous silica, and polystyrene nanoparticles, the method revealed a remarkably consistent hard corona thickness of approximately 50 nm, with particle‐level standard deviations ranging from 12 to 27 nm. Sensitivity analysis, localization precision assessment, control experiments, and day‐to‐day reproducibility measurements supported the robustness of the measurement strategy. Proteomic analysis showed similar molecular weight distributions across all nanoparticle classes, consistent with the comparable external surface chemistries used here.

## Graphical abstract

Quantitative dual‐color STORM imaging provides direct nanoscale measurement of protein coronas on nanoparticles in biological media. The approach reveals consistent ∼50 nm corona layers, establishing a reproducible method for characterizing the nanomaterial‐protein interfaces.

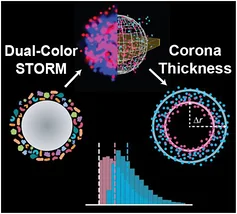

## 1 Introduction

Engineered nanomaterials have become important tools in biomedicine because their sizes, surface chemistries, morphologies, and pore structures can be tailored for applications including imaging, sensing, diagnostics, and drug delivery [1, 2, 3, 4]. Upon exposure to complex fluids such as blood plasma or serum‐containing culture media, biomolecules rapidly adsorb to the nanoparticle surface and redefine the interface presented to cells and tissues [5]. This adsorbed layer, referred to as the protein corona, governs how nanoparticles are recognized, trafficked, and processed in biological systems by altering their effective size, surface charge, colloidal stability, and accessibility of functional groups [6, 7, 8].

In complex media, many proteins compete for the nanoparticle surface, and the resulting layer evolves in a dynamic process as abundant, fast‐diffusing proteins adsorb first and are subsequently displaced or reorganized by less abundant but higher‐affinity species [9, 10]. This behavior leads to the definition of the “hard” and “soft” coronas [11, 12]. The soft corona contains weakly associated, dynamically exchanging proteins, whereas the hard corona contains proteins that are strongly associated with the nanoparticle surface and, in contrast to the soft corona, are typically retained after washing or isolation. Because the soft corona is transient and difficult to preserve without disturbing the interface, most research has focused on the hard corona. Even so, while the composition of the hard corona has been studied extensively, most available techniques to study the hard corona do not directly provide single‐particle structural measurements of corona thickness [13]. Hydrodynamic techniques such as dynamic light scattering (DLS) are often used as an initial indication of corona formation because an increase in hydrodynamic diameter is consistent with protein adsorption [9]. However, DLS provides an effective hydrodynamic diameter rather than a direct geometric shell thickness, and is sensitive to hydration, diffusion of the adsorbed layer, and especially aggregation [14, 15]. As a result, the same apparent increase in diameter can arise from a thin adsorbed layer, a more diffuse interaction zone, modest aggregation, or some combination of these effects. Imaging methods such as transmission electron microscopy (TEM) and cryo‐TEM can offer direct evidence of adsorbed protein layers, and have shown that nanoparticle coronas are often patchy, diffuse, and heterogeneous rather than perfectly uniform shells [16, 17]. These approaches are valuable because they reveal structural features that bulk methods cannot. At the same time, the method by which samples are prepared for these techniques can lead to molecular packing and constriction, reducing the measured corona thickness [18, 19]. In addition, they typically rely on 2D projections of a 3D interface, and quantitative thickness analysis can be challenging when the associated protein layer is low contrast, spatially irregular, or difficult to resolve across particles.

To address these limitations, we used stochastic optical reconstruction microscopy (STORM), a super‐resolution technique that exploits the photoswitching behavior of fluorophores to generate high‐resolution reconstructions from individual localization events [20, 21]. The fluorophores are sequentially photo‐switched on and off, and the position information of the resulting “blinks” is collected in a database, allowing for reconstruction and detailed examination. This information can be used to accurately measure protein locations on nanoparticle surfaces, facilitating the study of the protein corona. For example, we recently used STORM to map the penetration depth of proteins of various sizes within mesoporous silica nanoparticles (MSNs) [22]. This information was used to draw conclusions about how proteins compete for adsorption sites in a size‐restricted environment, which in turn helped to explain the accumulation of low‐molecular‐weight proteins in the coronas of MSNs. In another study, Feiner‐Gracia et al. used STORM to study the thickness of the protein corona created from a single protein [23]. Here, we show that dual‐color STORM can be used to measure the thickness of the protein corona formed in biologically relevant, complex protein solutions. This study focuses on establishing and validating the methodology across nanoparticle sizes, porosities, and materials, providing a structural foundation for subsequent investigations into the thermodynamics, kinetics, and biological fate of the protein corona.

Dual‐color STORM extends single‐channel reconstructions by simultaneously localizing two spectrally distinct fluorophores, enabling direct comparison of their nanoscale spatial distributions [24]. This capability has been widely applied in biological systems, such as resolving the nanoscale cytoskeletal architecture [25] or mapping the spatial organization of multi‐protein complexes at the nuclear envelope [26]. In materials research, dual‐color STORM has been used to visualize the interfaces within hybrid nanoparticle or polymer‐lipid assemblies [27, 28]. These applications highlight the unique ability of dual‐color STORM to reveal structural relationships that are inaccessible to single‐channel techniques. Here, the nanoparticle surface was labeled with a fluorophore emitting at 647 nm, and the hard corona was labeled with a fluorophore emitting at 488 nm. This allowed direct measurement of the radial separation between the nanoparticle surface and the protein layer adsorbed from complex serum. The resulting structural thickness is derived from the radial distributions of nanoparticle‐associated and protein‐associated localizations rather than a perfectly discrete molecular boundary. In this way, the present approach provides a direct measurement of the hard corona on individual nanoparticles and complements existing methods that primarily emphasize composition or bulk‐averaged changes in diameter.

## 2 Results and Discussion

### 2.1 Description of the Measurement Strategy

For our current experiments, we examined several parameters, including nanoparticle size, porosity, and composition. Dense (non‐porous) silica nanoparticles with diameters of 290, 680, and 840 nm were prepared by literature methods [29]; MSNs with diameters of 680 and 880 nm were prepared using a modified protocol described in a previous study [30]; and polystyrene nanoparticles with a diameter of 630 nm were purchased commercially. Each type of nanoparticle was functionalized with amines to provide reactive groups for conjugation with the far‐red fluorescent dye AZDye 647 TFP ester. The nanoparticles were then incubated in fetal bovine serum (FBS) in DMEM to form the protein corona, after which Atto 488 NHS ester was conjugated to the hard corona.

**SCHEME 1:**
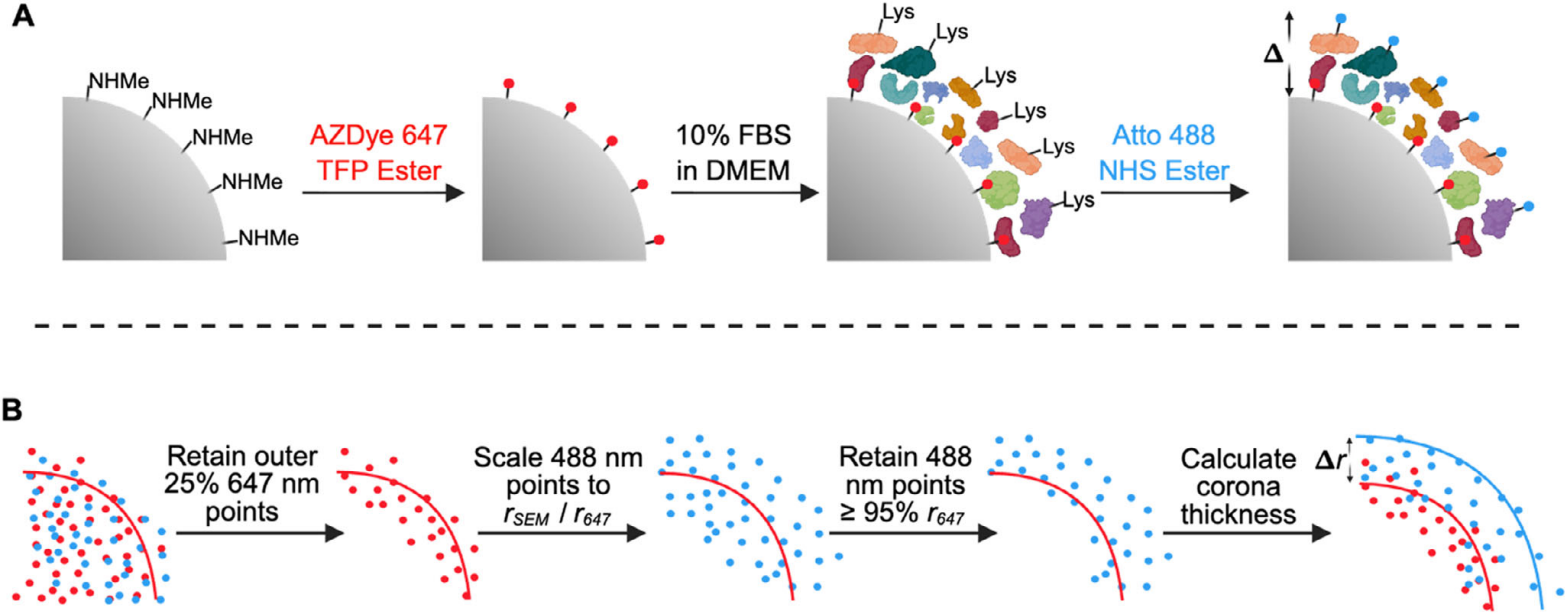
Experimental (A) and computational (B) procedures for dual‐color STORM measurements of protein corona thickness.

Dual‐color STORM was performed on the resulting particles by simultaneously acquiring data from the 647 and 488 nm channels: the former marked the nanoparticle surface, and the latter marked the outside of the corona. The thickness of the protein corona could therefore be determined by careful calculation of the nanoparticle radius in each channel, and then subtraction of the 647 nm radius from the 488 nm radius. The localization data (i.e., points of fluorescence emission) obtained from STORM were processed using the sequential workflow shown in Scheme 1.

**FIGURE 1:**
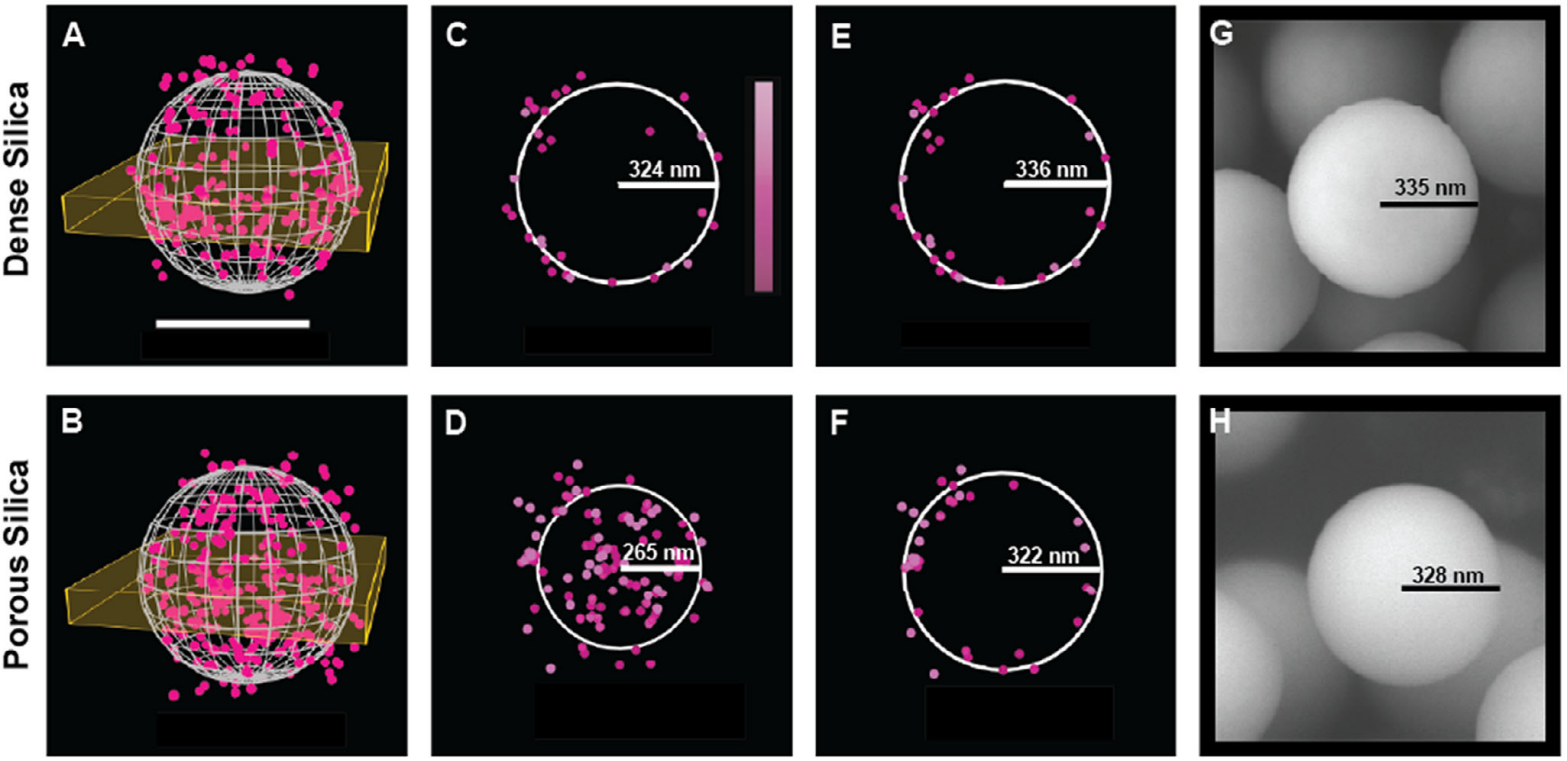
Defining nanoparticle radii using 647 nm channel localizations with comparison to FESEM images. Representative dense silica (top) and porous silica (bottom) nanoparticles are shown. (A, B) Wireframe reconstructions of 647 nm channel localizations with a 100 nm cross‐section in the center of the nanoparticle along the z‐axis are highlighted (yellow box). (C, D) Flattened localizations within these cross‐sections, plotted as a function of fluorescence intensity, with the calculated 647 nm channel radius outlined. (E, F) The same localizations after filtering to retain the outer 25% of 647 nm localizations, yielding radii consistent with expected particle sizes. (G, H) FESEM images of the respective particle types. Scale bar = 500 nm.

For each nanoparticle, localizations from the 647 and 488 nm channels were combined to define the nanoparticle center, determined as the mean position of all points after removing outliers, and coordinates were translated so the center was aligned with the origin. The channels were then analyzed separately. For the 647 nm channel, the nanoparticle radius (*r*_647_) was defined as the median 3D distance of the outer 25% of localizations. This minimized contributions from internal signal and fitting noise while preserving the external particle shell used as the structural reference surface. As shown in Figure 1, this procedure yielded nanoparticle radii consistent with values obtained from FESEM.

Localizations in the 488 nm channel were scaled by an adjustment factor (*r*_SEM_/*r*_647_) to correct for particle‐to‐particle variability, and only points located at radial distances ≥95% of *r*_647_ were retained to focus analysis on proteins at the outer edge of the corona rather than signal near or within the nanoparticle surface. This step was particularly important for porous silica, where proteins adsorbed within the nanoparticles. The corona boundary (*r*_488_) was then defined as the median of the retained 488 nm distances, and the corona thickness (*∆r*) was calculated as the difference between *r*_488_ and *r*_647_. This analysis was performed independently for 50 nanoparticles of each type, yielding per‐particle values for corona thickness. The thickness of the corona for each sample was then calculated from these particle‐level thickness measurements to reflect the overall behavior of each particle population.

### 2.2 Validation of Corona Thickness Measurements

Sensitivity analyses were performed on the mean thickness obtained from the full 50‐particle dataset for both dense and porous 680 nm silica samples (Table S1). Varying the fraction of 647 nm localizations used to define the particle boundary and changing the radial distance cutoff in the 488 nm channel used to define the outer edge of the corona each shifted the absolute mean thickness values, as expected for a boundary‐based measurement [31]. However, the selected 25% boundary definition and 95% cutoff produced values near the middle of the tested ranges rather than at an extreme. Inclusion or omission of the *r_SEM_/r_647_* scaling step also had only a modest effect on the final mean thickness values. Across all conditions, the calculated corona thickness remained in the same overall range beyond the nanoparticle surface.

Additional validation supported the quality of the single‐particle measurements themselves (Table S2). Mean lateral localization precision was approximately 10 nm for the 647 nm channel and 15 nm for the 488 nm channel across all particle types. These values fall within the range expected for dual‐color STORM on this length scale [32, 33] and are significantly less than the observed corona thicknesses of approximately 50 nm. Importantly, these values describe the uncertainty associated with individual localizations, whereas corona thickness was determined from the aggregate radial distributions of many localizations in each channel for a given particle. The final thickness measurement, therefore, reflects the relative positions of the nanoparticle‐associated and corona‐associated localization “clouds” rather than the uncertainty of any single localization. Measurements used our calculations typically included hundreds of localizations in the 647 nm channel and ∼100 localizations in the 488 nm channel, providing sufficient sampling for robust per‐particle radius estimation.

The procedure was also reproducible across independent experimental days (Table S3). For 680 nm dense silica, 680 nm porous silica, and 630 nm polystyrene particles, coronas were formed fresh on three separate days, and each set was then labeled, imaged, and analyzed independently. Across these replicates, the measured thickness remained within the same overall range for each particle type.

### 2.3 Single‐Particle Reconstructions Reveal Spatial Organization of the Corona

**FIGURE 2:**
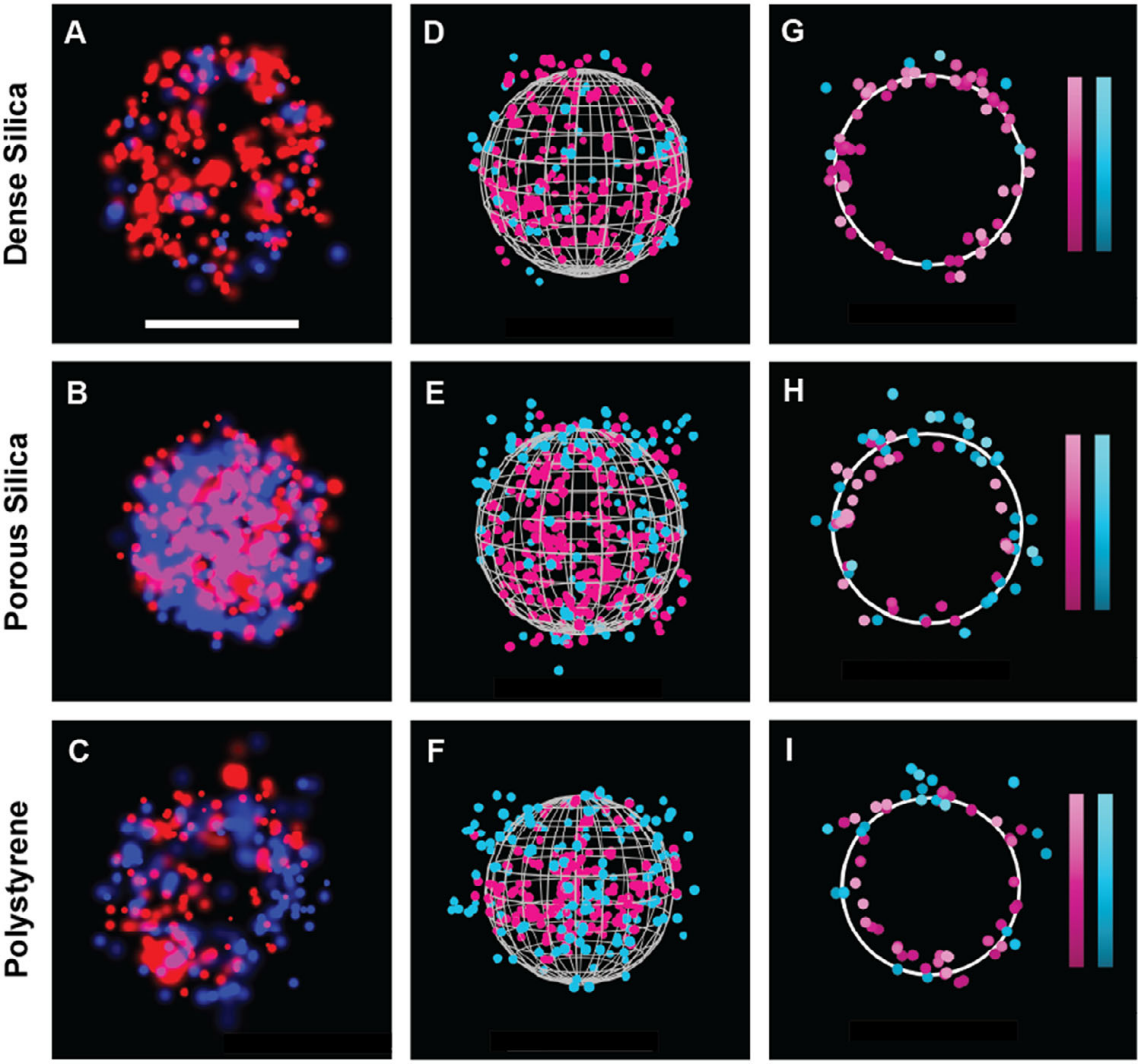
Representative dual‐color STORM analysis of protein coronas on dense silica (top), porous silica (middle), and polystyrene (bottom) nanoparticles. (A–C) NIS‐Elements STORM views of individual nanoparticles. (D–F) Reconstructed nanoparticle wireframes with fluorophore localizations from the 647 nm (magenta) and 488 nm (cyan) channels. (G–I) Flattened 2D cross‐sections generated by extracting data within 50 nm of the particle center along the z‐axis, plotted as a function of their fluorescence intensity (indicated in the colored bars). Scale bar = 500 nm.

Representative reconstructions of nanoparticles illustrate the structural features of the protein corona at the single‐particle level (Figure 2). Across all samples, the fluorophores emitting at 647 nm formed a clear image of a spherical nanoparticle upon reconstruction (shown in magenta), confirming consistent particle morphology and surface labeling. In contrast, the 488 nm emission (shown in cyan) displayed material‐dependent differences in spatial distribution. Prior to data filtering, dense silica particles showed a relatively uniform, well‐separated nanoparticle shell while maintaining a hollow interior, a feature indicative of non‐porous materials. Polystyrene particles exhibited noticeably more variability between particles, with more asymmetric coronas. The MSNs showed the densest and most spatially distributed 488 nm emission, with signal appearing throughout the particles and more tightly packed within the nanoparticle in both the single‐particle and cross‐section reconstructions. This is a result of increased protein adsorption near pore openings and within the nanoparticles. These distinct patterns, which cannot be captured by bulk techniques, highlight the advantage of using super‐resolution imaging to analyze the organization and density of the protein corona.

**FIGURE 3:**
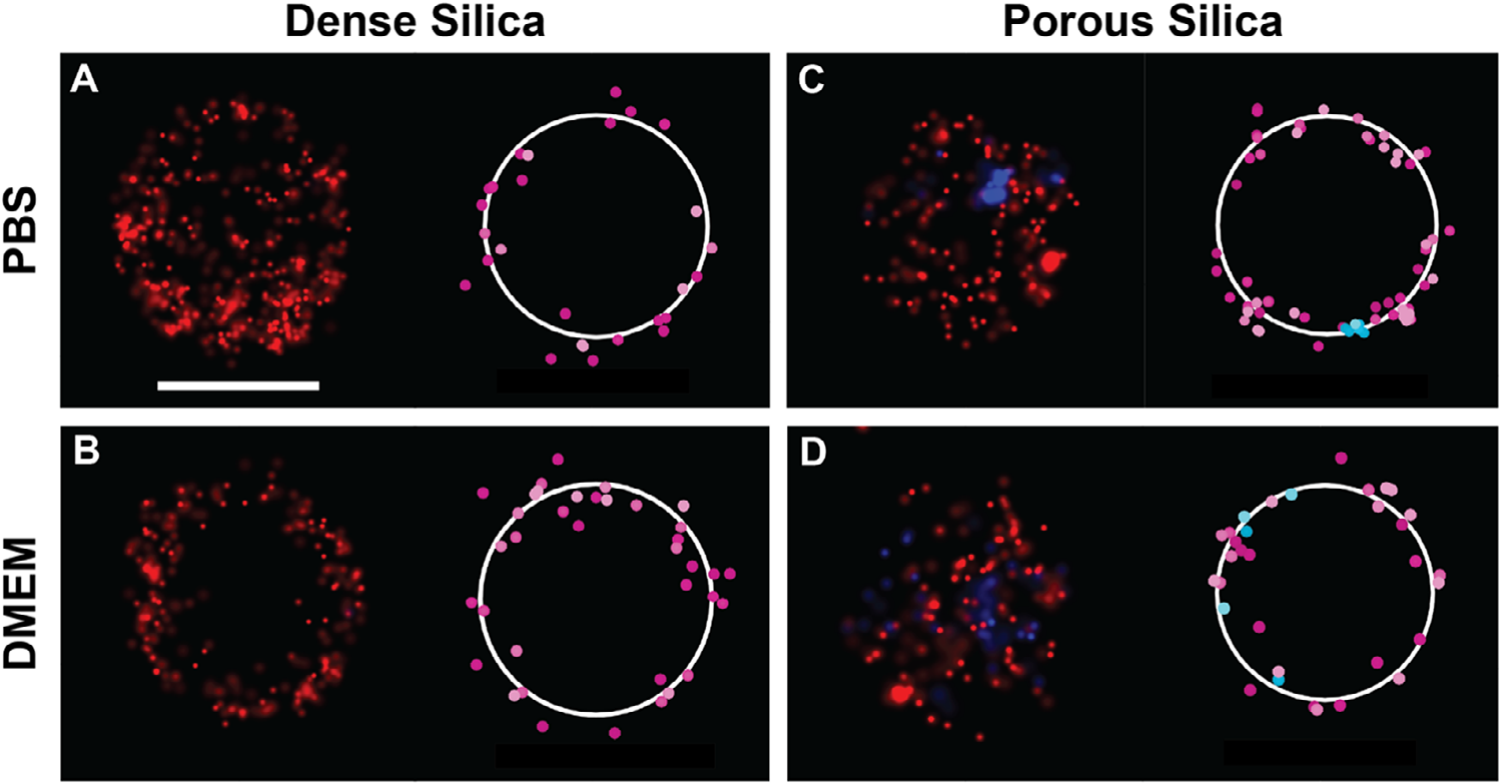
Representative STORM images and corresponding flattened 2D cross‐sections for (A, B) 680 nm dense silica nanoparticles and (C, D) 680 nm MSNs incubated in PBS or DMEM (i.e., not exposed to FBS and therefore without a protein corona). Scale bar = 500 nm.

Control experiments further supported assignment of the 488 nm signal to the protein corona. Dense and porous silica nanoparticles incubated in PBS or DMEM without FBS did not exhibit the same outer 488 nm shell observed after serum exposure (Figure 3). Although some 488 nm localizations were detected in the control samples for mesoporous silica (Table S4), they were sparse relative to the 647 nm channel and were not organized into the clearly separated exterior corona‐like distribution observed after incubation in FBS‐containing medium. This is consistent with the extremely large internal surface area of the MSNs, which results in some amines that remain unlabeled with 647 nm dye and could potentially bind 488 nm fluorophores. However, in the protein‐containing experiments, the pores are rapidly filled with protein before the 488 nm dye is applied, which minimizes binding to the MSN surface. Additionally, our procedure for processing the data removes internally bound fluorophores, and very few, if any, free amines remain on the external surface.

**FIGURE 4:**
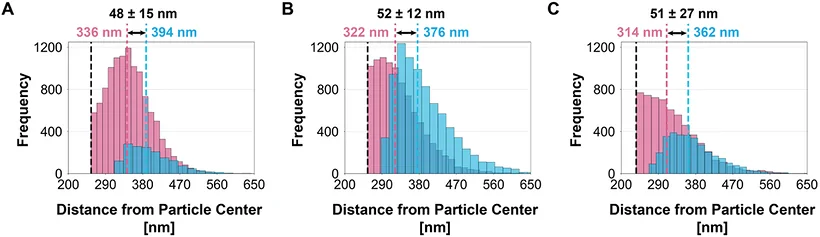
Radial distributions of STORM localizations: (A) dense silica, 680 nm; (B) porous silica, 680 nm; and (C) polystyrene nanoparticles, 630 nm. Histograms show pooled distances of all localizations from 50 particles per sample (magenta, 647 nm localizations, nanoparticle radius; cyan, 488 nm localizations, protein corona radius). The black dashed line marks the 25% retainment threshold applied to exclude the non‐surface 647 nm signal. Magenta and cyan dashed lines indicate the median distances across all localizations in each channel, and the difference between the two median distances is shown at the top of each plot.

Radial distance histograms were generated for each sample to evaluate the distribution of 647 and 488 nm emitting fluorophores (Figure 4). Across all particle types, the 647 nm channel showed symmetric distributions of fluorescence emission centered near the measured core radius, confirming consistent spherical labeling on the external surfaces of the particles regardless of type. In contrast, histograms from the 488 nm channel showed broader radial distributions, a quantitative representation of the qualitative images from STORM in Figure 2. This variability was most pronounced in the polystyrene sample, consistent with its higher standard deviation in thickness. Together, these reconstructions and radial distributions show that dual‐color STORM captures structural features of the hard corona that are not accessible from ensemble‐averaged measurements and provides the spatial basis for the particle‐level thickness analysis described below.

### 2.4 Particle‐Level Thickness Measurements Across Nanoparticle Types

**FIGURE 5:**
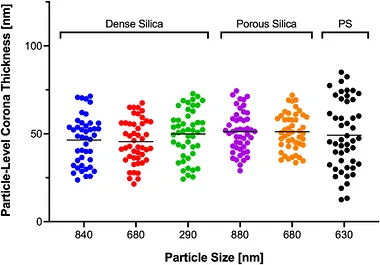
Particle‐level hard corona thicknesses measured by dual‐color STORM across six nanoparticle types. Each point represents one nanoparticle, and the horizontal bar indicates the mean thickness for that sample. Summary statistics are provided in Table 1. PS = polystyrene.

The particle‐level corona thicknesses are shown in Figure 5 and summarized in Table 1. Despite differences in particle diameter, porosity, and core material, all nanoparticle types clustered in the same overall ∼50 nm thickness regime. Polystyrene nanoparticles exhibited the greatest particle‐to‐particle spread, which may have been related to the somewhat heterogeneous distribution of the protein corona on those particles’ surfaces. Although the single‐particle measurements reveal clear particle‐to‐particle heterogeneity, that heterogeneity is distributed around a common structural length scale rather than around distinct material‐specific thicknesses.

**TABLE 1:** Summary of particle sizes, corona thicknesses, and statistical parameters.

	Particle Diameter [nm]	Mean Corona Thickness ± SD [nm]	Standard Error of the Mean [nm]	n
Dense Silica	840	49 ± 20	2.8	50
	680	48 ± 15	2.2	47
	290	51 ± 17	2.5	48
Porous Silica	880	52 ± 12	1.8	47
	680	52 ± 12	1.7	47
Polystyrene	630	51 ± 27	4.0	48

These measured thicknesses are substantially larger than the 10–20 nm values often inferred from DLS or reported from cryo‐TEM for silica and polystyrene nanoparticles in serum‐containing systems [15, 34]. In the case of DLS, corona thickness is inferred from the difference in hydrodynamic diameter before and after protein adsorption, where each mean particle size carries its own error and is sensitive to hydration, aggregation, and the inversion procedures used to extract size distributions [35]. The hydration of the hard corona can make its outer boundary quite diffuse and difficult to measure by this technique. Even modest polydispersity can broaden the apparent size distribution and introduce substantial uncertainty into shell‐thickness estimates [36]. Although STORM is also performed in a buffered solution, it is able to more accurately measure the diffuse outer boundary of proteins through fluorescent labeling and super‐resolution microscopy on individual nanoparticles. Cryo‐TEM, while valuable for visualizing adsorbed protein layers, requires preparation of samples by cryofixation, which can lead to molecular packaging and coating constriction. In cryofixation, the extreme cooling can reduce thermal motion and cause thermal contraction, resulting in more condensed molecular arrangements [18, 19]. Additionally, it relies on identifying a low‐contrast and often spatially irregular corona in 2D projection, which can make the full outer extent of the adsorbed layer difficult to define consistently [16]. For these reasons, the somewhat larger hard corona thickness measured here from dual‐color STORM can be explained in the context of the thinner coronas reported in the literature from DLS and cryo‐TEM.

The consistency of the measured thickness across nanoparticle classes is also mechanistically informative. For the dense silica series, substantial changes in particle diameter and curvature did not produce a detectable change in corona thickness. Likewise, the porous particles adsorbed more protein overall because of their larger accessible internal surface area, but once the pores were filled or blocked, the external corona thickness remained indistinguishable from that of the dense particles [37]. These observations suggest that, under the present conditions, the ultimate radial extent of the hard corona is not dictated primarily by modest differences in curvature or by the presence of internal porosity. Instead, the data are more consistent with a model in which the outer corona reaches a similar structural limit across these nanoparticle classes, likely governed more by the packing and organization of adsorbed serum proteins than by the underlying core once the initial adsorption layer has formed. This interpretation is consistent with literature showing that protein adsorption progressively redefines the nanoparticle interface and that the biological identity presented to the surrounding medium is increasingly determined by the adsorbed biomolecular layer rather than the pristine material surface [38, 39].

The observed spread in thickness values is also important to interpret. Because the measured lateral localization precision was substantially smaller than the final corona thickness values and the results remained robust across reasonable alternative processing choices, the particle‐to‐particle spread is most consistent with genuine heterogeneity in corona structure rather than methodological uncertainty alone [40]. At the same time, this conclusion should be interpreted within the scope of the present study. All particles shared similar amine‐functionalized external surface chemistry and were evaluated in the same serum‐containing medium, so the present data do not isolate material identity independently from surface chemistry or biological environment. Rather, the results support the conclusion that under a common surface chemistry and serum condition, the hard corona converges to a similar outer thickness across the nanoparticle classes examined here.

### 2.5 Proteomics

Proteomic analysis of the coronas formed on all six nanoparticle types (Figure S1) revealed similar molecular weight distributions, regardless of size, porosity, or material. In every case, proteins under 50 kDa comprised the majority of the corona composition, followed by progressively smaller fractions in the 50–70 kDa and 70–140 kDa ranges. Proteins above 140 kDa were consistently the least abundant. This pattern indicates that the size distribution of proteins in the corona is largely independent of particle physical properties under the present conditions. All particles were prepared with the same amine‐functionalized external surface chemistry and labeled with the same fluorophore, resulting in broadly comparable interfaces. This likely contributed to the similarity in molecular weight distributions across particle types, consistent with previous proteomics studies showing that corona composition is largely conserved across nanoparticle sizes and materials in complex biological media when surface chemistry is comparable [41].

## 3 Conclusion

This work establishes a robust, reproducible method for direct corona thickness measurement that will serve as the foundation for upcoming studies quantifying temperature‐ and time‐dependent corona formation and corona evolution under cell uptake‐mimicking conditions. Importantly, this study found that coronas formed in serum extend to approximately 50 nm in thickness, which is substantially larger than the 10–20 nm layers often inferred from bulk measurements. Because the method resolves particle‐to‐particle heterogeneity rather than relying on population‐averaged size shifts, it should be broadly useful for future studies of corona formation as a function of time, environment, and nanoparticle design. In this way, dual‐color STORM offers a complementary approach to existing hydrodynamic and compositional methods by adding spatial information that is otherwise difficult to obtain, thereby improving our ability to characterize how nanoparticles are structurally presented in biological media.

## 4 Materials and Methods

### 4.1 Materials

All chemicals were purchased from Sigma–Aldrich and used without modification unless otherwise stated. AZ‐Dyes were purchased from Vector Laboratories. ATTO dye was purchased from ATTO‐TEC. Amine‐modified polystyrene nanoparticles were purchased from CD Bioparticles. Dulbecco's Modified Eagle's Medium (DMEM) was purchased from Corning. OxyFluor was purchased from OxyRase. Fetal bovine serum was purchased from ThermoFisher Scientific.

### 4.2 Synthetic Procedures

#### 4.2.1 Synthesis of Dense 290 and 840 nm Silica Particles

Dense silica nanoparticles were synthesized by modifying the Stöber method [29]. The following procedure describes the synthesis and surface modification of 290 nm particles; Table S5 shows the adjustments made for different particle diameters. EtOH (100%, 46 mL) was mixed with NH_4_OH (13.7 m, 10 mL) in a 500 mL round‐bottom flask equipped with a magnetic stir bar. This mixture was allowed to thermally equilibrate before tetraethoxysilane (TEOS, 1 in 4 mL ethanol, 5 mL) was added to the solution. This mixture was then stirred for 2 h at room temperature. Afterward, the precipitate was isolated via centrifugation (14 800 rpm, 5 min) and resuspended in ethanol. This process was repeated two times to remove as much unreacted reagent as possible, and the resulting particles were dried under vacuum overnight.

#### 4.2.2 Synthesis of Dense 680 nm Silica Particles

Dense silica nanoparticles were synthesized by modifying the Stöber method [42]. EtOH (100%, 50 mL) was mixed with NH_4_OH (14.8 m, 10 mL) in a 250 mL round‐bottom flask equipped with a magnetic stir bar. This mixture was allowed to thermally equilibrate before tetraethoxysilane (TEOS, 0.5 in 2 mL ethanol, 2.5 mL) was added to the solution. This mixture was then stirred for 2 h at room temperature. Afterward, additional TEOS (7 in 28 mL ethanol, 35 mL) was slowly added to grow the particles to their desired size, and this new solution was stirred for 2 h. The precipitate was isolated via vacuum filtration and washed several times with ethanol. The resulting particles were dried under vacuum overnight.

#### 4.2.3 Synthesis of 680 and 880 nm Mesoporous Silica Nanoparticles (MSNs)

Mesoporous silica particles of 680 and 880 nm were synthesized according to a previously published protocol [43]. EtOH (100%, 276 g), Milli‐Q H_2_O (324 g), and NH_4_OH (14.8 m, 23.2 mL) were combined in a round‐bottom flask equipped with a magnetic stir bar. CTAB (0.546 g) was then added and allowed to completely dissolve under stirring at 1000 rpm and 750 rpm for 680 and 880 nm particles, respectively. After 5 min, TEOS (2.776 mL) was added, and the reaction proceeded for 2 h at room temperature. The resulting particles were then isolated via vacuum filtration, washed with water and ethanol, and dried under vacuum overnight.

#### 4.2.4 Modifying the External Surface of Dense Silica Nanoparticles With Amines

All three dense silica particle sizes followed a modified version of a previously published amine modification protocol [44]. The as‐prepared dense particles were resuspended in toluene (35 mL), followed by the addition of N‐methyl‐3‐aminopropyltrimethoxysilane (N‐MAPTMS, 166 µL). The solution was refluxed for 2 h. Once cooled to room temperature, the modified particles were isolated via filtration, washed three times with ethanol, and dried under vacuum.

#### 4.2.5 Modifying the External Surface of 680 and 880 nm MSN With Amines

External surface amines were grafted to unmodified MSN (MSN^+^‐OH) using a modified version of a previously published protocol [30]. Briefly, MSN^+^‐OH (840 mg) was added to dry toluene (12 mL) in a 25 mL round‐bottom flask equipped with a magnetic stir bar. The resulting solution was stirred at 300 rpm under nitrogen until the particles were fully resuspended (10 min). Aminopropyl triethoxysilane (APTES, 430 µL) was added, and the solution was allowed to stir at 250 rpm overnight at room temperature. The precipitate was isolated via centrifugation (10 000 x g, 1 min). The particles (MSN^+^‐NH_2_) were washed three times with toluene, twice with methanol, once more with toluene, and once with dichloromethane, each with thorough sonication to ensure complete resuspension.

To remove surfactant from the pores, MSN^+^‐NH_2_ (750 mg) were refluxed at 88°C in an ammonium nitrate/ethanol solution for 5 h. After reflux, the solution was cooled on ice, vacuum filtered, and washed three times with ethanol. The amine‐modified particles (MSN‐NH_2_) underwent an ion exchange using sodium chloride (1 m, 5 mL) to remove unwanted ammonium ions and were dried under vacuum.

### 4.3 Characterization

Nanoparticle morphology and size were determined by field emission scanning electron microscopy (FE‐SEM) using a ZEISS Gemini Sigma 300 VP microscope operating at 5 kV. Particles were adhered to 12 mm PELCO Tabs resting on aluminum specimen mounts and imaged under vacuum. Nitrogen gas physisorption isotherms were measured in a Micromeritics TriStar II PLUS apparatus. Samples were degassed at 110°C under nitrogen for 5 h prior to analysis. Surface area calculations were carried out using the BET method, and pore size and volume were calculated using the KJS adjustment of the BJH method [45]. Thermogravimetric analysis (TGA) was performed using a Pyris 1 TGA.

### 4.4 Particle Labeling, Corona Formation, and Corona Labeling

#### 4.4.1 Coupling Fluorophores With 647 nm Emission to the Nanoparticle Surface

Silica particles were resuspended in PBS (1x, pH 7.4, 1 mg/50 µL) and sonicated for 5 min. An aliquot of 250 µL containing 5 mg of particles was added to a separate tube with an additional 750 µL PBS. AZ‐Dye 647 TFP ester (5 µg) was then added, and the solution was allowed to shake at 1000 rpm for 1 h at room temperature. The fluorescently labeled particles were isolated via centrifugation (10 000 rpm, 1 min) and washed two times with PBS. 1 mg aliquots were prepared by resuspending the nanoparticle pellet in 1 mL PBS, briefly sonicating, and pipetting 200 µL into new tubes. The particles were stored at 4°C and protected from light until use.

Polystyrene particles underwent a similar process with a slight change in preparation. The stock bottle obtained from CD Bioparticles was used to make a working particle solution (10 mg/mL in PBS, 1 mL). To this working solution, 20 µg AZ‐Dye 647 TFP ester was added. From there, the polystyrene particles followed the same procedure as the silica particles.

#### 4.4.2 Formation of the Protein Corona

To form the protein corona, 1 mg of nanoparticles obtained above was resuspended in 100–200 µL PBS (1x, pH 7.4), briefly sonicated for 10 min, and added to 1 mL of 10% FBS in DMEM. The nanoparticle‐protein solution was incubated on an Eppendorf Thermomixer C1.5 set at 1000 rpm and 23°C for 1 h. Following incubation, the particles were pelleted by centrifugation at 5000 x g for 1 min and washed 2–3 times with PBS to remove unbound proteins.

#### 4.4.3 Coupling Fluorophores With 488 nm Emission to the Protein Corona

To fluorescently label the adsorbed proteins after corona formation, the particle‐protein pellet was resuspended in PBS (1 mL), and ATTO488 NHS Ester was added (5 µg for dense and polystyrene particles, 0.5 µg for porous particles). The solution was mixed on a laboratory rocker at room temperature for 30 min, followed by centrifugation and two washes of PBS to remove unreacted dye.

### 4.5 STORM Preparation, Imaging, and Data Analysis

#### 4.5.1 dSTORM Imaging

Samples were resuspended in 1 mL PBS (1x, pH 7.4), briefly sonicated, and 100 µL of suspension was plated onto 35 mm MatTek Glass Dishes previously cleaned with piranha solution (3:1, H_2_SO_4_: H_2_O_2_). After 3 min, the particle solution was removed, and the dish was gently washed twice with PBS to remove loosely adhered particles. Fresh imaging buffer was prepared immediately prior to imaging and added carefully to the dish. The buffer consisted of sodium lactate (100 µL), mercaptoethylamine‐HCl (MEA‐HCl, 50 µL, 500 mm), Oxyfluor (15 µL), sodium hydroxide (5 µL, 5 m), and PBS (1x, pH 7.4, 330 µL).

Dual‐color STORM imaging was performed on a Nikon Eclipse Ti‐E inverted microscope equipped with an Andor iXON3 DU897 EMCCD camera. A quarter‐wave plate and cylindrical lens were inserted into the light path to enable simultaneous imaging of the 647 and 488 nm channels. Images were collected over a 256 × 256‐pixel region of interest with an exposure time of 1 frame. A total of 10 000 frames were acquired per channel, with an average imaging time of 5 min per sample.

Before export of localization lists, ND2 files were processed using the NIS‐Elements Run STORM Analysis software and the standard instrument calibration routines for dual‐color 3D STORM. A minimum peak height threshold of 300 was applied for localization detection in both channels. Channel alignment and chromatic aberration correction were performed using bead‐based calibration files generated on the microscope prior to sample analysis, including the standard z‐calibration and XY registration/warp correction used for multicolor acquisition. These corrections were applied before downstream analysis so that the relative spatial positions of the 647 and 488 nm channels reflected the calibrated dual‐color reconstruction rather than uncorrected channel offsets. Individual nanoparticles were then isolated manually, and the corresponding localization molecule lists for the 647 and 488 nm channels were exported as txt files. Particles selected for analysis were well separated from neighboring particles and free of obvious large aggregates or overlapping reconstructions that would interfere with particle‐level radius determination. Approximately 50 nanoparticles were analyzed per condition.

#### 4.5.2 Image Analysis and Reconstruction

Exported localization lists were processed using a custom‐built Python analysis pipeline written in Python 3.11 with the Pandas, NumPy, SciPy, and Matplotlib libraries. For each nanoparticle, localizations from the 647 and 488 nm channels were first combined to compute a shared particle center. Prior to center determination, extreme localization outliers were removed iteratively from the 3D coordinate set using a distance‐based filtering procedure. The remaining coordinates were then translated so that the shared particle center was aligned with the origin.

After center translation, the two channels were processed separately. For the 647 nm channel, radial distances were calculated from the translated coordinates and filtered using an interquartile range (IQR)‐based criterion. A physically motivated lower bound of 75% of the expected particle radius was then applied to emphasize the external nanoparticle shell and reduce contributions from internal signal or fitting noise. The nanoparticle radius, *r_647_*, was defined as the median of the retained 647 nm radial distances.

For the 488 nm channel, radial distances were likewise IQR‐filtered and then normalized on a per‐particle basis using the factor *r_SEM_/r_647_* to account for modest particle‐to‐particle variation in measured nanoparticle radius. Only 488 nm localizations at radial distances greater than or equal to 95% of *r_647_* were retained for corona analysis. This step emphasized the outer protein‐associated signal and was especially important for mesoporous silica particles, where protein‐associated localizations could also appear closer to the particle interior. The corona radius, *r_488_*, was defined as the median of the retained 488 nm distances, and corona thickness for each particle was calculated as *∆r = r_488_ – r_647_*.

Auxiliary outputs generated by the analysis procedure, including normalized radial‐distance files, xyz coordinate files, and z‐filtered cross‐sections, were used for visualization and quality control but were not used directly in the primary thickness calculation.

#### 4.5.3 Validation and Reproducibility of the Measurement Strategy

Several features of the procedure were evaluated to assess method robustness. Sensitivity analyses were performed by varying key processing parameters, including the fraction of 647 nm localizations used to define the nanoparticle boundary, the radial cutoff applied to the 488 nm localizations, and the use of per‐particle scaling for the 488 nm channel. These analyses were used to assess whether the final thickness values were stable across reasonable alternative processing choices.

Localization precision values were extracted from the NIS‐Elements Run STORM analysis outputs for each particle channel and summarized on a per‐particle basis as an internal measure of localization quality. These values represent the software‐reported lateral (XY) localization accuracy, which is internally calculated using a Thompson‐type formula based on the detected photon counts. Reproducibility was evaluated using independently prepared 680 nm dense silica, 680 nm porous silica, and 630 nm polystyrene samples across three separate experimental days, with corona formation, post‐corona labeling, imaging, and computational analysis repeated using the same procedure described above.

### 4.6 Proteomics Analysis

#### 4.6.1 Digestion of the Protein Corona

Nanoparticles containing the corona were suspended in digestion buffer (36 µL, 50 mm ammonium bicarbonate, 0.25 mm urea, 4% acetonitrile) and reducing buffer (6 µL, 100 mm dithiothreitol in H_2_O). The resulting solutions were allowed to incubate for 1 h at 70°C. After 1 h, the alkylation buffer was added, and the samples were incubated at room temperature for 20 min, covered from light. Protein digestion was achieved by adding trypsin (15 µL, 40 ng/µL) and incubating overnight at 37°C. To stop the digestion process, formic acid (15 µL, 10%) was added. The solution was centrifuged, and the supernatant was removed. The resulting digested sample was desalted using a ZipTip C_18_ (P100, Pierce, Thermo Scientific) according to the manufacturer's protocol and dried in a SpeedVac.

#### 4.6.2 Liquid Chromatography‐Tandem Mass Spectrometry (LC‐MS/MS)

Peptide digests were resuspended in a solution of 2.5% MeCN and 2.5% FA in LC‐MS grade water for subsequent chromatographic separation and mass spectrometry analysis on an Orbitrap Exploris 240 coupled to an EASY‐nLC 1200 (Thermo Scientific, Waltham, MA, USA). Samples were loaded onto a 100 µm x 300 mm capillary column packed with 1.8 µ 100A Magic3 C18AQ stationary phase (Premier LC‐MS, CA, USA) and separated at a flow rate of 300 nL/min at 700 bar using a mobile phase mixture of two solvents A: 100% water/0.1% FA and B: 80% MeCN/0.1% FA with a gradient of 5%–30% B over 25 min, 30%–50% B over 20 min, 50%–95% B over 15 min, then 95% B for 9 min followed by a hold at 5% B. Peptides were introduced to the mass spectrometer via nanospray ionization (NSI) with a spray voltage of 1900 V and ion transfer tube temperature of 300°C. Mass spectrometry data were collected in data‐dependent acquisition mode. Survey scans from 350–1400 m/z at 60 000 resolution and tandem mass spectrometry scans on the most abundant ions with an HCD collision energy at 30% at 15 000 resolution and an isolation window of 2 m/z were collected over a cycle time duration of 3 s.

#### 4.6.3 Database Searching

The files generated from each chromatographic run were imported into Proteome Discoverer 3.1 (Thermo Fisher Scientific). Tandem mass spectra were searched using the SEQUEST node in the processing procedure against a bovine UP000009136 FASTA database downloaded from Uniprot and processed using the Minora Feature Detector to perform precursor quantification based on peak intensities normalized to the total peptide amount. Search parameters included specifying full trypsin enzymatic activity and setting dynamic modification on methionine residues (+15.9949 Da: oxidation) and static modification on cysteines (+57.021 Da: Carbamidomethyl). The Percolator node was added to the processing procedure to set the false discovery rate to less than 1%.

#### 4.6.4 Molecular Weight Binning

Normalized precursor intensities provided by the proteomics facility were used as input for molecular weight (MW) binning. Proteins were assigned to one of four MW bins: <50 kDa, 50–70 kDa, 70–140 kDa, and >140 kDa. For each biological replicate, the normalized intensities were summed within each MW bin and expressed as a percentage of the total intensity for that replicate. These replicate‐level percentages were then averaged across triplicates for each nanoparticle type, and the standard deviation was calculated to provide error bars in the graphical representation.

### 4.7 Statistical Analysis

Corona thickness was calculated at the individual particle level as described in Section 4.5. Preprocessing included iterative removal of extreme localization outliers during particle‐center determination, followed by channel‐specific filtering of radial localization distributions prior to radius determination. For the 647 and 488 nm channels, radial distances were filtered using an interquartile range (IQR)‐based criterion before application of the channel‐specific boundary definitions. Additional cutoffs were then applied, including a lower‐bound cutoff for the 647 nm channel and retention of only 488 nm localizations at radial distances greater than or equal to 95% of *r_647_* for corona‐boundary determination. For most IQR‐based preprocessing steps in this procedure, an IQR multiplier of 1.5 was used. The exception was the final filtering of particle‐level mean corona thickness values at the sample‐summary stage, where an iterative IQR filter with a multiplier of 1.0 was applied.

Data are presented as mean ± standard deviation (SD) unless otherwise noted. Median, IQR, and standard error of the mean (SEM) were additionally calculated for selected datasets. The sample size, *n*, corresponds to the number of individual nanoparticles included in a given particle‐level analysis after filtering.

Data processing and statistical calculations were performed using custom scripts written in Python with Pandas, NumPy, and SciPy. Plotting and figure preparation were performed using GraphPad Prism, Python script, and Blender, as appropriate.

## Conflicts of Interest

The authors declare no conflict of interest.

## Supporting information




**Supporting File**: smtd70709‐sup‐0001‐SuppMat.pdf.

## Data Availability

The data that support the findings of this study are available from the corresponding author upon reasonable request.
