# Supplementary material for: Direct Measurement of Protein Corona Thickness on Nanomaterials Using Dual‐Color Stochastic Optical Reconstruction Microscopy (STORM)
Source: Small Methods. 2026 May 11;10(16):e02394. doi: 10.1002/smtd.202502394 (PMC13505707; doi:10.1002/smtd.202502394)
Supplement: Supplementary file 1 [file SMTD-10-e02394-s001.pdf]

Supporting Information

Direct Measurement of Protein Corona Thickness on Nanomaterials Using Dual-Color Stochastic Optical Reconstruction Microscopy (STORM)

*Bradley T. Cech, Sophie M. Sullivan, and Christopher C. Landry**

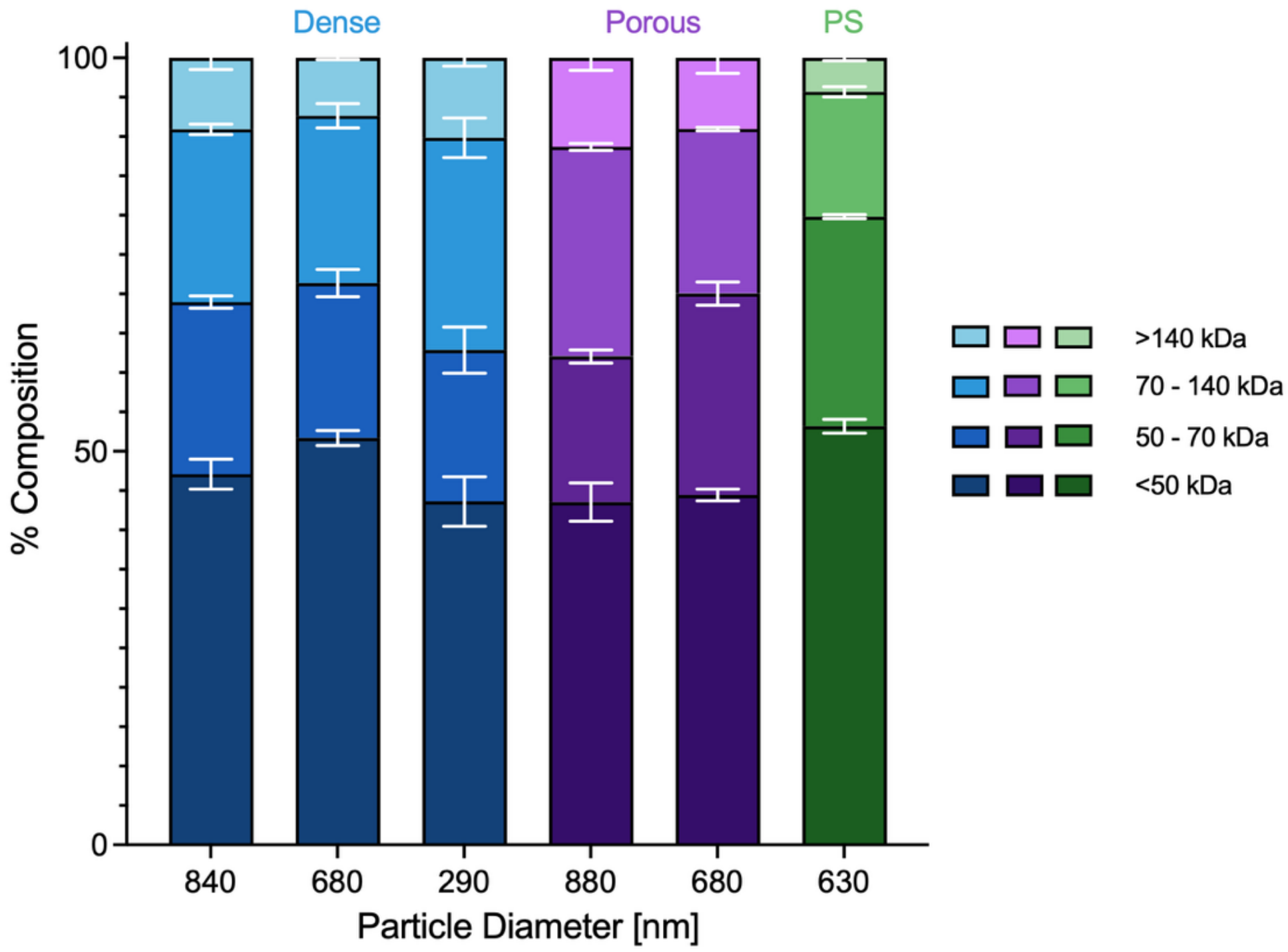

Figure S1. Proteomic analysis of corona composition across six nanoparticle types. Bar graphs show the percentage of total protein abundance assigned to four molecular weight bins. Data represent the mean of three biological replicates per particle type, with error bars showing standard deviations.

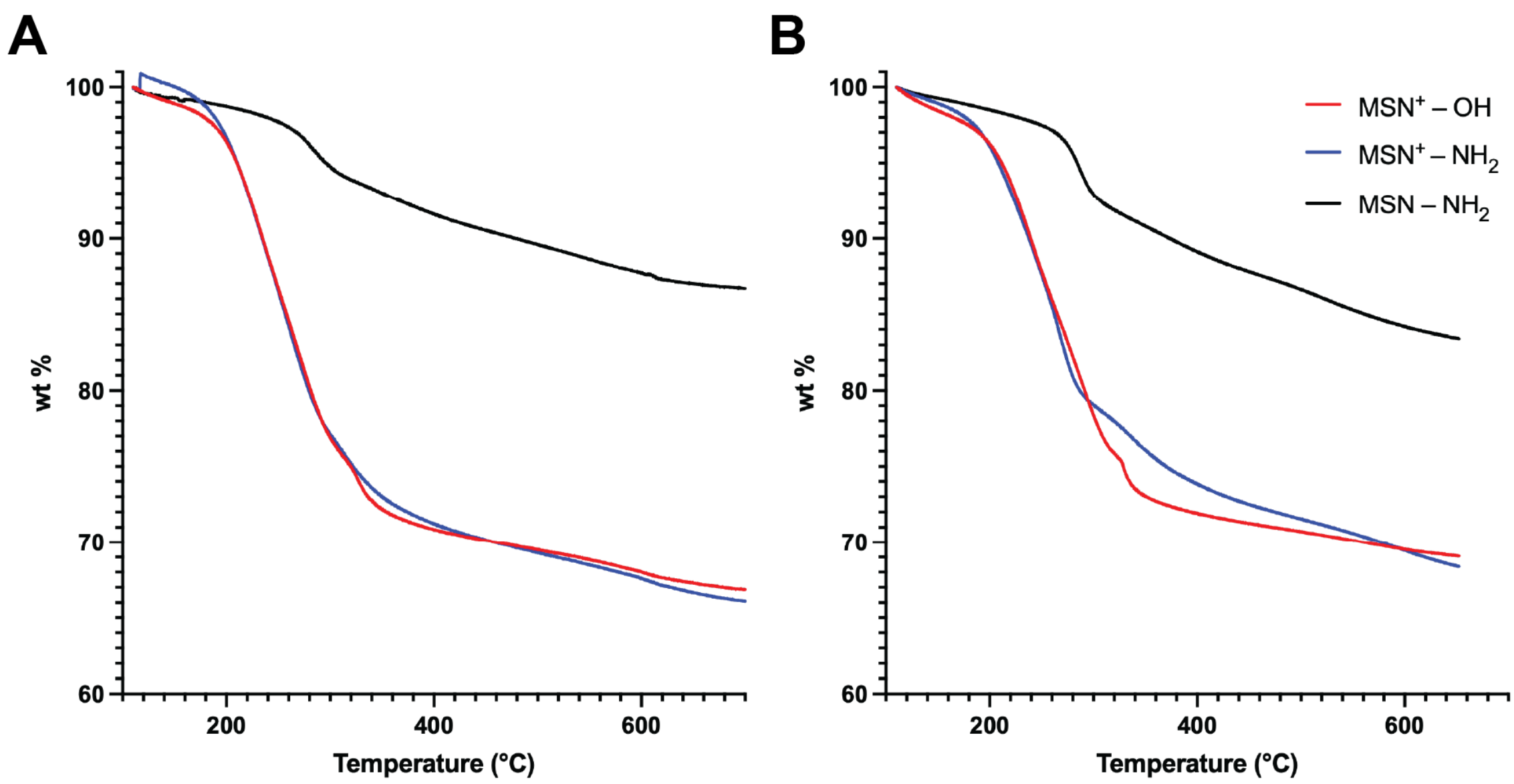

Figure S2. TGA weight loss curves of (A) 680 nm and (B) 880 nm MSNs during surface modification. Data normalized to 110 ºC highlight surfactant removal.

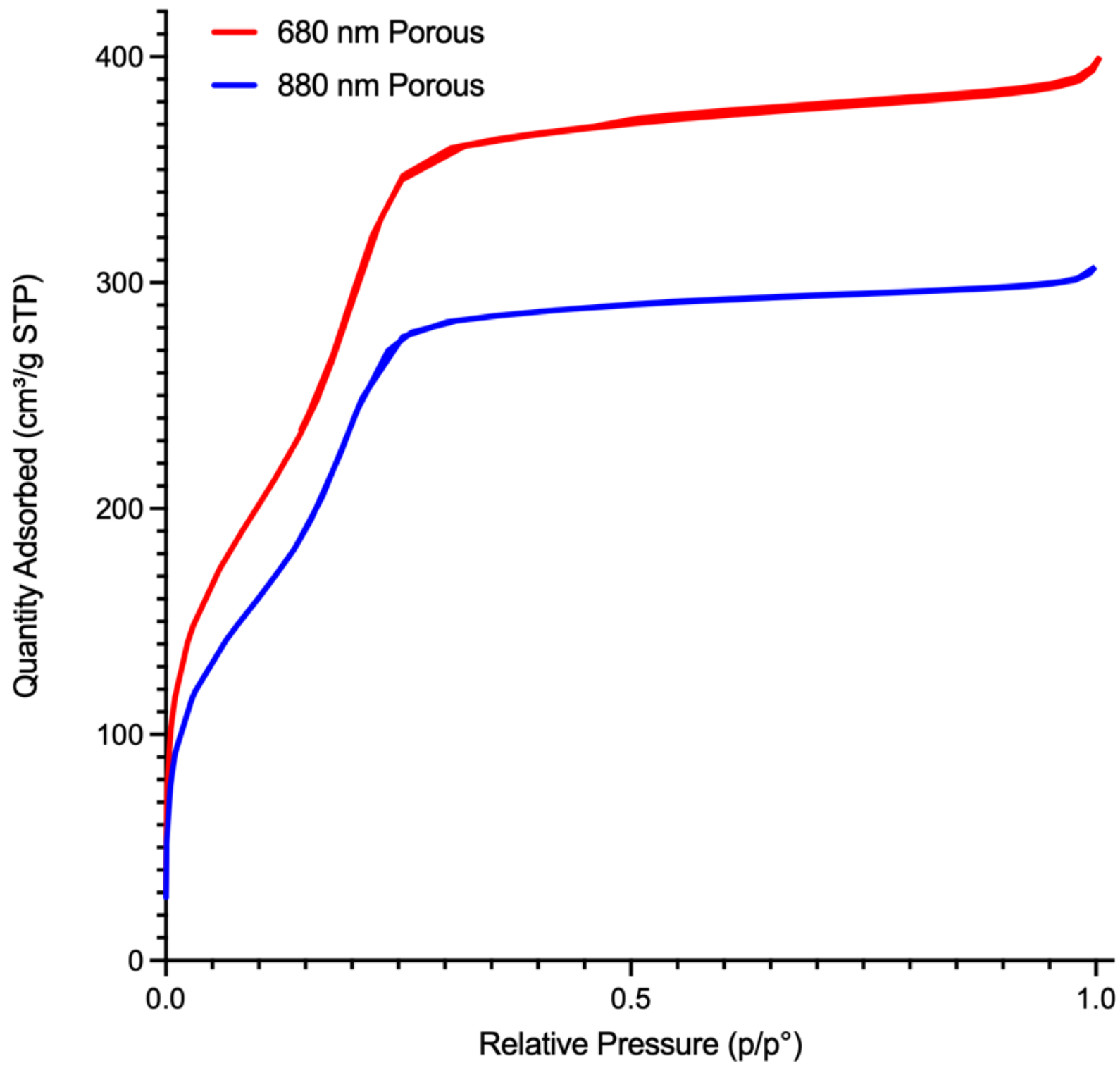

Figure S3. N_2 physisorption isotherms of 880 nm (blue) and 680 nm (red) MSNs. Corresponding results are summarized in **Table S6**.

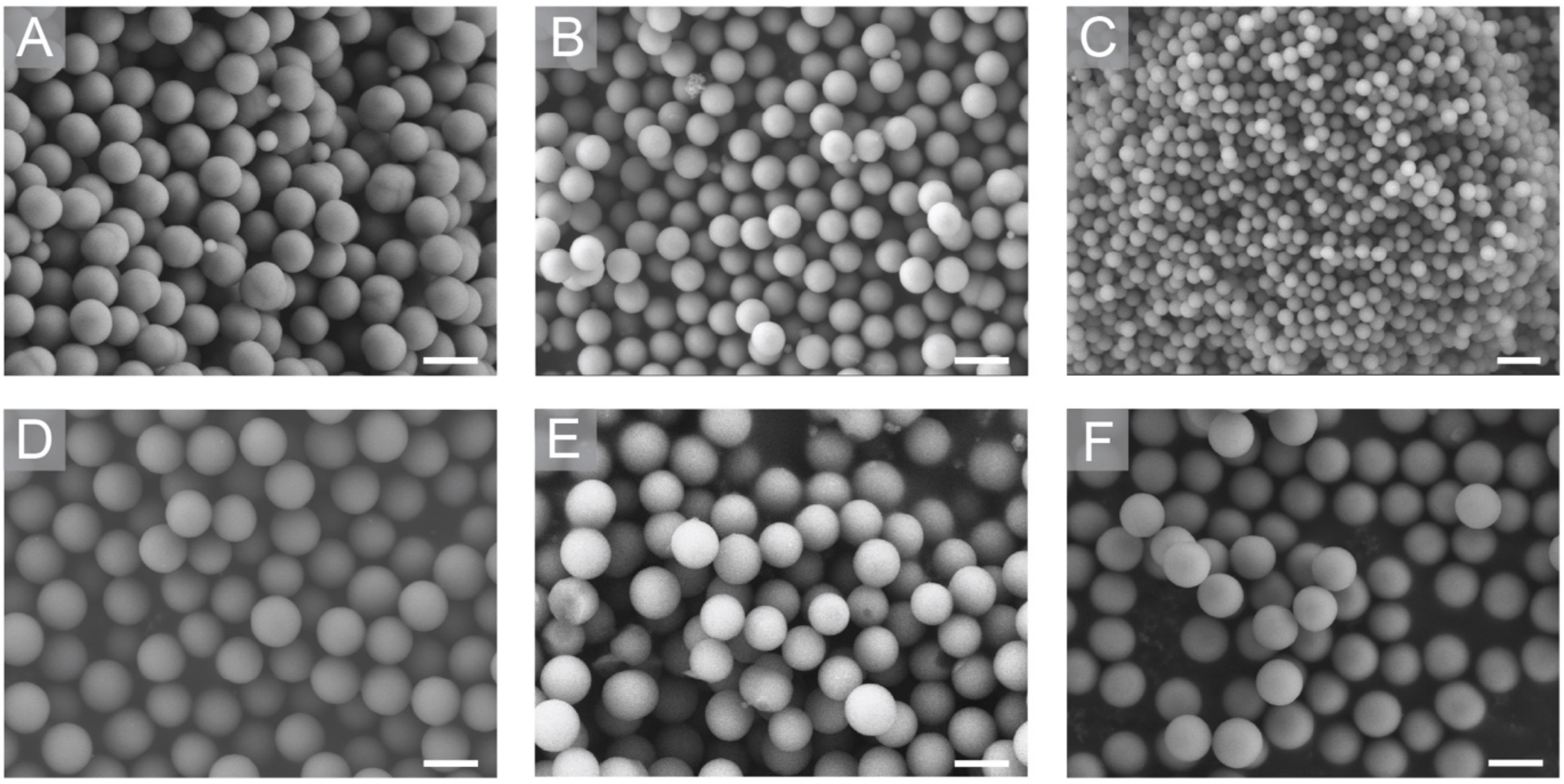

Figure S4. FESEM images of dense silica nanoparticles and MSNs. (A) 840 nm dense silica; (B) 680 nm dense silica; (C) 290 nm dense silica; (D) 880 nm MSNs; (E) 680 nm MSNs; and (F) 630 nm polystyrene nanoparticles. Scale bars = 1 μm.

Table S1. Sensitivity analysis of the dual-color STORM thickness measurements of the protein corona for representative dense and porous silica datasets.

	Parameter Varied	Condition	Mean Hard Corona Thickness ± SD [nm]	n
**Dense Silica, 680 nm**	647 nm boundary	outer 35%	55 ± 17	47
		outer 30%	51 ± 14	46
		outer 25%	48 ± 15	47
		outer 20%	42 ± 12	44
		outer 15%	36 ± 9	40
	488 nm cutoff	≥ 90% r_{647}	40 ± 17	47
		≥ 92.5% r_{647}	43 ± 17	49
		≥ 95% r_{647}	48 ± 15	47
		≥ 97.5% r_{647}	51 ± 15	47
		≥ 100% r_{647}	57 ± 14	47
	488 nm scaling	off	50 ± 16	49
		on (r_{SEM}/r_{647})	48 ± 15	47
**Porous Silica, 680 nm**	647 nm boundary	outer 35%	70 ± 16	48
		outer 30%	61 ± 13	47
		outer 25%	52 ± 12	47
		outer 20%	43 ± 11	45
		outer 15%	36 ± 9	44
	488 nm cutoff	≥ 90% r_{647}	43 ± 14	47
		≥ 92.5% r_{647}	48 ± 12	47
		≥ 95% r_{647}	52 ± 12	47
		≥ 97.5% r_{647}	55 ± 11	44
		≥ 100% r_{647}	59 ± 10	42
	488 nm scaling	off	52 ± 12	47
		on (r_{SEM}/r_{647})	52 ± 12	47

Table S2. Summary of lateral localization accuracy and localization counts per particle. Lateral localization accuracy values were first averaged within each particle for each channel, and the reported values correspond to the mean ± SD of these per-particle averages across the analyzed particles. Localization counts are reported as the mean ± SD per particle.

	**Particle Diameter [nm]**	**647 nm Lateral Localization Accuracy [nm]**	**488 nm Lateral Localization Accuracy [nm]**	**647 nm Localizations per Particle**	**488 nm Localizations per Particle**
**Dense Silica**	840	11.0 ± 0.5	16.8 ± 1.1	487 ± 100	78 ± 32
	680	9.5 ± 0.5	15.1 ± 1.1	242 ± 47	46 ± 14
	290	11.4 ± 0.5	19.4 ± 1.8	358 ± 133	39 ± 20
**Porous Silica**	880	9.8 ± 1.0	11.4 ± 1.2	295 ± 85	126 ± 68
	680	10.0 ± 0.8	14.7 ± 1.1	306 ± 93	293 ± 101
**Polystyrene**	630	10.1 ± 0.8	16.0 ± 1.1	179 ± 72	111 ± 47

Table S3. Interday reproducibility of hard corona thickness measurements for representative nanoparticle classes.

	**Experimental Day**	**Mean Thickness ± SD [nm]**	**Standard Error of the Mean [nm]**	**n**
**Dense Silica, 680 nm**	1	48 ± 15	2.2	47
	2	50 ± 13	1.9	47
	3	51 ± 14	2.0	48
**Porous Silica, 680 nm**	1	52 ± 12	1.7	47
	2	52 ± 14	2.1	47
	3	47 ± 17	2.5	49
**Polystyrene, 630 nm**	1	51 ± 27	4.0	48
	2	51 ± 29	4.1	50
	3	50 ± 18	2.6	50

Table S4. Summary of localization counts per particle for the control samples. Localization counts are reported as the mean ± SD per particle.

	Incubation Medium	647 nm Localizations per Particle	488 nm Localizations per Particle
Dense Silica Control, 680 nm	PBS	417 ± 230	3 ± 3
	DMEM	285 ± 69	1 ± 1
Porous Silica Control, 680 nm	PBS	155 ± 73	26 ± 18
	DMEM	110 ± 46	27 ± 24

Table S5. Synthesis conditions for dense silica particles.

Particle Diameter [nm]	EtOH [mL]	NH_4OH [mL]	TEOS [mL]	Time [h]	Stir Rate [rpm]
840	184	40 [13.7 M]	4 + 16 mL EtOH	2	300
680	50	10 [14.8 M]	0.5 + 2 mL EtOH<br>7 + 28 mL EtOH	4	1000
290	46	10 [13.7 M]	1 + 4 mL EtOH	2	1000

Table S6. N_2 physisorption data for MSNs.

Particle Diameter [nm]	BET Surface Area [m^2 g^{-1}]	Average Pore Diameter [nm]	Total Pore Volume [cm^3 g^{-1}]
880	1,065	2.3	0.46
680	1,304	2.3	0.60
